# Behavioural intervention to increase physical activity in adults with coronary heart disease in Jordan

**DOI:** 10.1186/s12889-016-3313-5

**Published:** 2016-07-26

**Authors:** Eman Alsaleh, Richard Windle, Holly Blake

**Affiliations:** 1 School of Nursing, Philadelphia University, Amman, Jordan; 2 School of Health Sciences, University of Nottingham, Queen’s Medical Centre, Nottingham, NG7 2HA UK

**Keywords:** Coronary heart disease, Physical activity, Intervention, Behavioural, Self-efficacy

## Abstract

**Background:**

Patients with coronary heart disease often do not follow prescribed physical activity recommendations. The aim of this study was to assess the efficacy of a behavioural intervention to increase physical activity in patients with coronary heart disease not attending structured cardiac rehabilitation programmes.

**Methods:**

Parallel randomised controlled trial comparing 6-month multi-component behavioural change intervention (*n* = 71) with usual care (*n* = 85) was conducted in two hospitals in Jordan, Middle East. Intervention included one face-to-face individualised consultation, 6 telephone support calls (for goal-setting, feedback and self-monitoring) and 18 reminder text messages. Patients were randomly allocated to the two groups by opening opaque sealed sequence envelopes. The patients and the researcher who provided the intervention and assessed the outcomes were not blinded. Outcomes were assessed at baseline and 6 months. Primary outcome was physical activity level, secondary outcomes were blood pressure, body mass index, exercise self-efficacy for exercise and health-related quality of life.

**Results:**

Intervention and control groups were comparable at baseline. Moderate physical activity significantly increased in the intervention group compared with control group (mean change (SD) of frequency: 0.23 (0.87) days/week versus -.06 (0.40); duration: 15.53 (90.15) minutes/week versus −3.67 (22.60) minutes/week; intensity: 31.05 (105.98) Metabolic equivalents (METs) versus 14.68 (90.40) METs). Effect size was 0.03 for moderate PA frequency, 0.02 for moderate PA duration and 0.01 for moderate PA intensity. Walking significantly increased in the intervention group compared with control group (mean change (SD) of frequency: 3.15 (2.75) days/week versus 0.37 (1.83) days/week; duration: 150.90 (124.47) minutes/week versus 24.05 (195.93) minutes/week; intensity: 495.12 (413.74) METs versus14.62 (265.06) METs). Effect size was 0.36 for walking frequency, 0.05 for walking duration, 0.32 for walking intensity and 0.29 for total PA intensity. Intervention participants had significantly lower blood pressure, lower body mass index, greater exercise self-efficacy and better health-related quality of life at 6 months compared with controls.

**Conclusions:**

Multi-component behavioural intervention increases physical activity, and improves body composition, physiological and psychological outcomes in CHD patients not attending structured rehabilitation programmes.

**Trial registration:**

Current Controlled Trials retrospectively registered in 21-03-2012. ISRCTN48570595.

## Background

Regular physical activity (PA) is recommended for coronary heart disease (CHD) patients for its role in the prevention and treatment of CHD risk factors such as hypertension and overweight [1–3] and positive benefits for quality of life [4, 5]. However, PA levels are consistently low among CHD patients [3, 6–9]. Inaccessibility or a lack of availability of PA programmes is an important factor in adherence to PA in CHD patients [10–12]. In Jordan, which is a developing country, challenges within the healthcare systems mean that the availability of structured PA rehabilitation programmes is poor [13]. Many Jordanian CHD patients do not adhere to general PA recommendations [13, 14] and report factors influencing engagement in PA which include low self-efficacy (or confidence) for PA, perceived barriers to PA and low motivation to be active [14, 15].

Behavioural change interventions have shown efficacy in increasing PA levels in CHD patients and improving psychosocial health [16–18], and often include strategies such as goal-setting, self-monitoring and feedback. Goal-setting has been found to be more effective when goals are short-term [19–21], specific [22–24] and set by patients or in collaboration with healthcare professionals (rather than by them) [25]. Self-monitoring using diaries or records has been found to be useful in raising awareness about existing behaviour [26] and increasing PA [4]; regular follow-ups and contacts by healthcare professionals are thought to enhance patients’ self-monitoring of goal achievements [19–21].

Studies have demonstrated the value of delivering behavioural change strategies through face-to-face consultation, and telephone follow-up [4, 27]. Mobile phone text messages are increasingly used to support healthcare, and have shown benefits for increasing PA in other settings [28]; text message reminders may therefore provide a useful mechanism for low-cost provision of regular patient contact.

In practice, behavioural change strategies are not implemented widely in either supervised or non-supervised PA interventions with CHD patients [4]. Whilst previous behavioural change interventions have shown some efficacy in increasing PA, research studies have been criticised for failing to provide sufficient detail about the behavioural change strategies employed, and the interventions have been criticised for lacking focus on addressing individual patients’ needs and a lack of provision of regular feedback to patients on their progress [18, 27, 29–32].

This study aimed to examine the efficacy of a behavioural change intervention to increase PA in Jordanian patients with CHD, through building self-efficacy (or confidence) for exercise, addressing barriers to exercise and motivating health behaviour change through tailored goal setting, feedback, monitoring and reminders. The efficacy of the intervention in improving secondary outcomes was assessed, including: blood pressure (BP), body mass index (BMI), exercises self-efficacy and health-related quality of life (HRQL). This paper reports the components of the intervention, the results of the primary and secondary outcomes, the discussion of the findings and the implication of the intervention in the practice and research.

## Methods

### Study design

This was a multicentre randomised controlled trial, and parallel group study. Consolidated Standards of Reporting Trials (CONSORT) statement guidelines were adopted in conducting and reporting the trial [33]. Ethical approval for the study was granted in the UK by the local institutional review board, and in Jordan by the institutional review boards at both participating hospital sites (Jordan University Hospital and King Abdullah University Hospital).

### Participants

The target population were Jordanian outpatients with CHD, that met the following eligibility criteria: a) were clinically stable and able to perform PA; b) were adults between the ages of 18 and 70 years; c) had access to a mobile telephone and; d) had no co-morbidities or unstable major health problems that prevented them from participation in PA. Sample size calculations indicated that a total sample size of 156 participants was required accounting for up to 15 % loss to follow-up based on a conservative estimate taken from previous studies [22, 27]. Statistical power was calculated based on the difference in mean change of PA amount (minutes per week) between the control and intervention groups as identified in previous studies [34]. This was to detect improvements in PA level of 30 min per week, which was deemed to be a moderately large clinically relevant difference between the control and intervention group [34]. This had an estimated standard deviation of 60 with a two-sided 5 % significance level and a power of 80 %.

### Procedure

Potential participants were identified from hospital records by the researcher and screened for eligibility. Consulting physicians were asked to confirm whether eligible participants were medically stable and capable of performing PA at the recommended level advised for the general population and the majority of CHD patients at the time of the study (30–60 min of moderate intensity PA on most, or preferably all days of the week). Once eligibility was confirmed, the researcher approached suitable patients in the outpatient clinics and provided them with study information sheets containing contact details for the researcher. Interested participants then contacted the researcher who arranged a suitable time to take informed consent at the hospital clinic, following which they were recruited into the study and baseline data collected. Recruitment and baseline data collection took place between February and March 2012.

Recruited participants were randomised to one of two groups. Randomisation was undertaken using prepared opaque sealed envelopes, by a researcher who was not involved in recruitment or the delivery of the intervention. Group 1 received usual care from their physicians, which consisted of general (rather than tailored) advice about the benefits of PA and instructions to engage in moderate-intensity PA, such as brisk walking. Group 2 received usual care, plus a six-month behavioural intervention delivered by a cardiac nurse. The intervention was delivered to all participants allocated to treatment by a single cardiac nurse who had training in lifestyle advice, and prior expertise in delivering health education and interventions using motivational interviewing techniques with cardiac patients. This ensured that the intervention was delivered consistently to all participants (with respect to length and duration of contacts and extent of advice given), and ensured intervention fidelity.

The intervention was delivered between February and August 2012. The ultimate objective of the intervention was to encourage participants to meet recommendations for daily PA at the time of the study; to engage in ‘*moderate PA for at least 30 min on most, preferably all, days of the week*’ [35, 36]. The intervention was informed by Social Cognitive Theory and Self-Efficacy Theory [37, 38] and adopted behavioural change strategies (goal-setting, self-monitoring, feedback) that were aimed at increasing PA levels. In an initial face-to-face consultation lasting 20–30 min, the cardiac nurse used motivational interviewing techniques to discuss and address patients’ barriers to PA, highlight perceived facilitators of active lifestyles, increase self-efficacy for PA and work collaboratively with them to set goals (initiated by the patient). Patients were encouraged to self-set achievable goals that were specific, individualised and short-term. Efforts to enhance self-efficacy involved highlighting patients’ achievements of their goals (performance accomplishment), providing feedback on their goal achievements (verbal encouragement), providing patients with encouragement statements on their progress in achieving their goals (vicarious experience) and helping patients to identify their positive perceptions about PA and correct their negative thoughts about PA (physiological and emotional status in capabilities). Patients were encouraged to self-monitor their PA levels through regularly recording their PA in a diary. Each patient then received six telephone call-based consultations with the same nurse (15–20 min each, one per month). In these consultation calls, the nurse provided tailored feedback on patients’ engagement in PA, reviewed their goals and diary notes and addressed any arising barriers to patients achieving their goals. Although nurse contact was relatively brief per contact, the frequency and duration of face-to-face and telephone call consultations was determined based on published evidence from studies with healthy people and patients with CHD (Furber et al., [27]; Hughes et al., [30]; Rodrigues et al., [18]). During the intervention period, patients received prompts and reminders to be physically active and meet personal goals, which were sent by mobile phone text message from a no-reply study telephone number. Text message content was based on reminding patients to perform and maintain the required level of PA and encouraging them to address barriers to PA. In addition, the text messages were informed by the Theory of Planned Behaviour (TPB) [39, 40]; targeting attitudes, subjective norms, perceived behavioural control and intention. In total, 18 reminder text messages were delivered to each patient at a rate of two messages every week in the first 3 months and one message every week in the last 3 months; since interventions using individualised or decreasing frequency of messages may be more successful than interventions using a fixed message frequency [41].

**Fig. 1:**
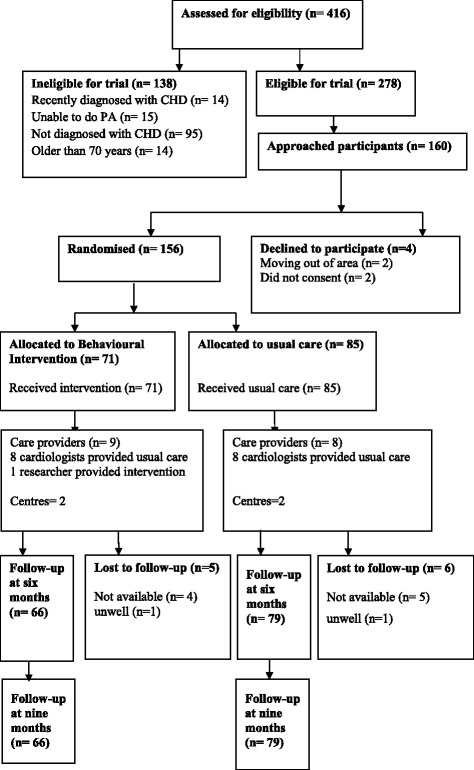
Flow of participants through the study

Outcome data were collected at two time points: baseline (immediately after recruitment) and immediately post-intervention (6 months after randomisation). The researcher collected questionnaire measures via structured interview, and at the same appointment, collected physiological and body composition measures. Three months after the end of the intervention (9 months after randomisation), participants in the intervention group were invited to complete a questionnaire survey to assess their perceptions of the intervention, current engagement in physical activity (meeting PA guidelines, or not) and PA intentions. Flow of participants through the study is provided in Fig. 1.

## Measures

### Socio-demographic and health information

The questionnaire included socio-demographic items (age, gender, marital status, living status and area, educational level, job status, income), items relating to CHD diagnosis and duration and co-morbidities, together with general health items (single item measures of diet, smoking behaviour and general health perception). Standardised questionnaire measures included PA, self-efficacy for exercise and HRQL. Physiological and body composition measures included blood pressure, height and weight.

### Questionnaire measures

#### Physical activity

Self-reported moderate PA and walking was measured using the short format of the International Physical Activity Questionnaire [IPAQ, 42] which has established reliability and validity [43]. This questionnaire contains four domains of PA: leisure time, domestic and gardening, work and transport related PA, and includes specific types of PA: walking, moderate-intensity and vigorous-intensity activity. Items relating to vigorous PA were not included since the CHD patients were not encouraged to perform PA at this level. Individuals reported their level of PA by writing the frequency (days per week) and duration (minutes per day) of each type of activity. To measure intensity of PA, metabolic equivalents (METs) were calculated.

#### Self-efficacy for exercise

Exercise self-efficacy was assessed using the Exercise Self-Efficacy Scale (ESES) which has established reliability and validity [44]. The scale incorporates nine items that describe situations in which people might experience difficulties in engaging in regular PA. Respondents rated their perceived ability to perform moderate physical activity in a range of circumstances, from 0 (not confident) to 10 (very confident). The total score was obtained by summing the numerical rating for each answer and dividing by the number of answers.

#### Health-related quality of life

HRQL was measured by the Mac-New Heart Disease HRQL [45] which has established validity and reliability, and has been shown to be acceptable to patients with CHD [46, 47]. This 27-item questionnaire evaluates the QOL aspects of physical, emotional and social functioning affected by CHD. Items are presented on a Likert scale ranging from one (indicating low HRQL) to seven (indicating high HRQL) [45].

### Physiological outcomes and body composition

#### Diastolic & systolic blood pressure

Blood pressure was measured by the researcher in the cardiac clinic with an automated electronic blood pressure monitor, using criteria determined by the American Heart Association [48], the British Hypertension Society [49] and the Medicines and Health Care Products Regulatory Agency [50]. The same monitor was utilised for all patients at baseline and at six months. A validity test was undertaken by taking four readings alternating with five mercury sphygmomanometer readings [51]. Both systolic and diastolic readings taken were at least within 5 mmHg of each other for at least 50 % of readings.

#### Body mass index

BMI was calculated from weight and height using the following formula: BMI = [weight in kilograms/[height in metres x height in metres]]. Individuals were classified into categories according to their BMI (< 18.5 = underweight, 18.5–24.9 = normal weight, 25–29.9 = overweight, > 30 = obese).

### Data analysis

Statistical analysis was conducted using SPSS for Windows version 20.0. Independent *t*-test was used to measure baseline to six-month changes of PA and secondary outcomes in both groups, and mean difference of mean changes of PA levels and secondary outcomes between control and intervention groups. Complete case analysis was utilised since missing data was considered to be ‘missing completely at random’ (MCAR). Intention to treat analysis (ITT) was adopted including multiple imputation and last observation carried forward. Independent *t*-test and chi-squared test were used to compare the demographic profile of the intervention with control group at baseline, and responders with non-responders at 6-month follow-up.

## Results

One hundred and fifty six participants were randomly allocated to the control (*n* = 85) and intervention groups (*n* = 71). Sociodemographic and health characteristics were not significantly different between the control and intervention groups at baseline (see Table 1). Study participants were comparable with the population from which they were drawn in terms of age, gender and medical diagnosis. All intervention participants (*n* = 71; 100 %) completed all six months of the intervention; low rates of loss to follow-up were observed (see Fig. 1).

**Table 1:** Sociodemographic and health characteristics

Characteristics	Control (*n* = 85)	Intervention (*n* = 71)	*p*
Age	58 ± 8.7	57.7 ± 10.5	0.49
Gender			
Male	43 (50.6)	41 (57.7)	0.42
Female	42 (49.4)	30 (42.3)	
Monthly income			
< 99 Jordanian dinars	6 (7.1)	6 (8.5)	0.60
100–299 Jordanian dinars	28 (32.9)	26 (36.6)	
300–499 Jordanian dinars	30 (35.3)	18 (25.4)	
> 500 Jordanian dinars	21 (24.7)	21 (29.6)	
Education level			
Primary	28 (32.9)	29 (40.8)	0.79
Secondary	21 (24.7)	14 (19.7)	
University	33 (38.8)	26 (36.6)	
None	3 (3.5)	2 (2.8)	
Marital status			
Single	5 (5.9)	5 (7)	0.73
Married	70 (82.4)	61 (85.9)	
Divorced	1 (1.2)	1 (1.4)	
Widowed	9 (10.6)	4 (5.0)	
Living status			
Alone	6 (7.1)	4 (5.6)	0.78
With spouse	4 (4.7)	5 (7)	
With spouse & children	75 (88.2)	62 (87.3)	
Job status			
Working	21 (21.7)	20 (28.2)	0.81
Not working	45 (52.9)	34 (47.9)	
Retired	19 (22.4)	17 (23.9)	
Living area			
City	75 (88.2)	60 (84.5)	0.63
Town	4 (4.7)	6 (8.5)	
Village	6 (7.1)	5 (7)	
Current CHD			
Chest pain	16 (18.8)	8 (11.3)	0.24
Catheterization	59 (69.4)	54 (76.1)	
Cardiac surgery	10 (11.8)	7 (9.9)	
Myocardial infarction	0.0 (0.0)	2 (2.8)	
Duration of diagnosis	61.5 ± 60.8	48.3 ± 45.3	0.13
Chronic disease			
Diabetes	6 (7.1)	8 (11.3)	0.63
Hypertension	33 (38.8)	22 (31.0)	
Diabetes & hypertension	29 (34.1)	24 (33.8)	
None	17 (20)	17 (23.9)	

Values are mean values ± SD or numbers and percentages in parenthesis

Sixty Five (92 %) of intervention participants completed at least one goal in the PA diary, and 66 (93 %) completed diaries for all 12 weeks of the intervention period. Adherence to completion of physical activity diaries dropped 3 % across the intervention period. Diary content and accomplishment of goals was not formally measured as an outcome, but was discussed within telephone consultations with the nurse as part of the process of tailored feedback and goal-setting.

### Physical activity

Participants were classified as physically active according to IPAQ when they met the PA guidelines of performing 30 min of moderate intensity PA five days a week (150 min per week), or through a combination of walking and moderate-intensity activities they achieved a minimum of at least 600 METs - minutes/week. There were no significant differences in PA at baseline, between groups. One quarter of the sample met PA recommendations at baseline (intervention: *n* = 17, 24 %; control: *n* = 22, 26 %). The proportion of participants meeting recommendations for daily PA significantly increased from baseline to six months in the intervention group (baseline: *n* = 17, 24 %; six months: *n* = 58; 88 %) and decreased in the control group (baseline: *n* = 22, 26 %; six months: *n* = 19; 24 %). Mean scores for moderate PA and walking levels (including frequency, duration and intensity) significantly increased from baseline to six-months in the intervention group but not in the control group. The difference in mean change of walking and moderate PA levels between the two groups was high. Mean change (SD) and mean difference (95 % confidence intervals) are shown in Table 2.

**Table 2:** Change in physical activity levels between control and intervention groups

PA levels	Control (*n* = 79)		Intervention (*n* = 66)		Mean change (SD)		Mean difference (95 % CI)
Moderate PA	Baseline *mean (SD)*	6 months *mean (SD)*	Baseline *mean (SD)*	6 months *mean (SD)*	Control group (*n* = 79)	Intervention group (*n* = 66)	
Frequency	0.45 (1.31)	0.39 (1.27)	0.70 (1.67)	0.93 (1.85)	−.06 (0.40)	0.23 (0.87)	−0.29* (−0.51 to −0.07)
Duration	20.13 (62.50)	16.46 (58.73)	21.14 (44.95)	36.67 (109.76)	−3.67 (22.60)	15.53 (90.15)	−19.20* (−39.98 to −1.58)
Intensity walking	77.97 (243.13)	63.29 (227.41)	83.18 (178.02)	114.23 (212.60)	−14.68 (90.40)	31.05 (105.98)	−45.73* (−77.97 to −13.50)
Frequency	3.03 (2.55)	3.40 (2.36)	3.0 (2.37)	6.15 (1.26)	0.37 (1.83)	3.15 (2.75)	−2.78* (−3.57 to −2.00)
Duration	84.81 (90.98)	108.86 (198.02)	86.97 (99.11)	237.88 (121.10)	24.05 (195.93)	150.90 (124.47)	−126.86* (−179.96 to −73.76)
Intensity	275.16 (300.71)	289.78 (302.74)	276.52 (301.63)	771.64 (396.44)	14.62 (265.06)	495.12 (413.74)	−480.61* (−597.61 to 363.61)
Total intensity	353.14 (415.29)	353.08 (406.88)	359.70 (598.27)	886 (426.52)	−0.06 (271.33)	526.30 (394.12)	−526.24* (−639.91 to −412.78)

PA: physical activity, frequency was measured by days/week, duration was measured by minutes/week, intensity was measured by METs/week, *: significant difference when *P*< 0.05

### Secondary outcomes

#### Health-related quality of life

HRQL increased from baseline to six-months in the intervention group but not in the control group. There was a significant difference between intervention and control groups in total HRQL and all three domains including physical, social and emotional domains. The difference in mean change of HRQL between the two groups was high. Mean change (SD) and mean difference (95 % confidence intervals) are shown in Table 3.

**Table 3:** Changes in secondary outcomes between control and intervention groups

Health variables	Control (*n* = 79)		Intervention (*n* = 66)		Mean change (SD)		Mean difference (95%CI)
	Baseline *M (SD)*	6 months *M (SD)*	Baseline *M (SD)*	6 months *M (SD)*	Control group (*n* = 79)	Intervention group (*n* = 66)	
SBP (mm Hg)	138.82 (18.20)	139.39 (14.06)	134.92 (17.59)	128.80 (14.27)	.57 (11.47)	−6.12 (10.79)	6.69* (3.01 to 10.37)
DBP (mm Hg)	78.16 (9.88)	82.96 (6.48)	75.71 (10.89)	73.92 (8.68)	4.80 (7.82)	−1.79 (5)	6.59* (4.46 to 8.71)
Body weight (K gm)	84.50 (31.21)	84.66 (32.20)	84 (30.20)	78.70 (21.31)	0.16 (11.20)	−5.30 (10.32)	5.46* (2.24 to 8.52)
Body mass index (kg/m^2^)	30.29 (6.09)	30.74 (5.50)	29.38 (5.40)	27.52 (5.47)	0.45 (2.41)	−1.85 (2.14)	2.30* (1.54 to 3.06)
HRQL (1–7)	3.85 (1.05)	3.70 (1)	4.21 (0.83)	5.30 (0.70)	−0.15 (0.60)	1.09 (0.66)	−1.24* (−1.45 to −1.03)
Emotional domain (1–7)	4.04 (1.13)	3.90 (1.11)	4.51 (0.96)	5.59 (0.67)	−0.14 (0.59)	1.08 (0.78)	−1.22* (−1.45 to 0.99)
Social domain (1–7)	3.98 (1.19)	3.80 (1.11)	4.26 (0.87)	5.20 (0.78)	−0.18 (0.73)	0.94 (0.68)	−1.12* (−1.36 to −0.89)
Physical domain (1–7)	3.50 (1.18)	3.38 (1.09)	3.82 (0.92)	4.93 (0.86)	−0.12 (0.77)	1.11 (0.75)	−1.23* (−1.48 to −0.98)
Exercise self-efficacy (1–10)	3.35 (1.69)	3.33 (1.67)	3.44 (1.62)	7.21 (0.88)	−0.02 (0.89)	3.77 (1.56)	−3.79* (−4.23 to −3.37)

*SBP* systolic blood pressure, *DBP* diastolic blood pressure, *HRQL* health related quality of life, *: significant difference when *P*<0.05

#### Self-efficacy for exercise

Exercise self-efficacy scale increased from baseline to six-months in the intervention group but not in the control group. The difference in mean change of exercise self-efficacy between the two groups was high. Mean change (SD) and mean difference (95 % confidence intervals) are shown in Table 3.

#### Physiological outcomes and body composition

##### Systolic & diastolic blood pressure

There was a significant decrease in mean value of SBP and DBP from baseline to six- months among the intervention group but not in the control group, indicating a significant difference in the mean change of SBP and DBP between intervention and control groups (Table 3).

##### Weight and body mass index

Mean body weight and BMI decreased significantly from baseline to six-months among the intervention group but not among the control group, indicating a significant difference in the mean change of body weight and BMI between intervention and control groups (see Table 3). The majority of patients in both intervention and control groups were overweight or obese at baseline (84.5 % and 87 % respectively); at six months there was a significant difference in the distribution of body weight between the two groups (*p* = 0.007). At six months there were more patients with normal body weight and fewer who were overweight or obese in the intervention group compared with those in the control group (Table 4).

**Table 4:** The distribution of body weight based on BMI at baseline and six months between the two study groups

Classification of Body weight	Baseline		*P*	Six months		*P* value
	Control group (*n* = 85)	Intervention group (*n* = 71)	0.47	Control group (*n* = 79)	Intervention group (*n* = 66)	0.007*
Underweight (< 18 kg/m^2^)	(0.0)	1 (1.4)		0.0 (0.0)	2 (3)	
Normal weight (18.5–24.9 kg/m^2^)	11 (12.29)	10 (14.4)		4 (5.1)	16 (24.2)	
Overweight (25–29.9 kg/m^2)^	32 (37.6)	26 (36.6)		32 (40.5)	23 (34.8)	
Obese (> 30 kg/m^2^)	42 (49.4)	34 (47.9)		43 (54.4)	25 (37.9)	

### Participants’ perceptions of the intervention

Of the intervention participants, *n* = 66 (92.96 %) completed the post-intervention evaluation survey (three months after the intervention ended). Perceptions of the intervention were positive, with 100 % reporting that they valued all elements of the intervention, including goal-setting, self-monitoring and feedback and delivery methods including face-to-face consultation, telephone call consultations and text messages. Participants reported multiple benefits of the intervention, which included: gaining knowledge about how to make behavioural changes, receiving regular reminders and encouragement which motivated them to be more active, and feeling supported by and building a trusting relationship with the cardiac nurse. A minority of participants (*n* = 13) raised barriers to being active after the intervention; these included being in poor health, not having enough time and not enjoying PA. The majority of participants reported that they had overcome their barriers to PA through engaging with the intervention, learning and implementing behavioural change strategies and increasing their knowledge about the importance of PA for their health.

At nine months, all those participants that were engaging in recommended levels of PA at six months (*n* = 58, 88 %) indicated that they had maintained this level of PA over the previous three months; of the inactive remainder, 9 % (*n* = 6) reported a positive intention to increase their PA levels.

## Discussion

This behavioural intervention was effective in increasing PA levels among patients with CHD and helping them to achieve the internationally recommended levels of daily PA required to benefit their health. The high recruitment rates, low attrition rates and positive feedback from intervention participants demonstrate the attractiveness and acceptability of this theory-based behaviour change intervention to CHD patients in Jordan.

The intervention was highly accessible to CHD patients. Firstly, it did not require their attendance at a supervised PA program. Secondly, the prescribed PA was walking which is a safe yet effective form of physical activity which can be undertaken without supervision and is therefore suitable for CHD patients [35, 52].

Findings are limited by self-reported measures of physical activity. Although the IPAQ is a valid and reliable measure of PA, self-reports, may give a distorted impression of PA levels (usually an overestimation) compared to objective measures of PA [53, 54]. However, standardized interviews were conducted when administering the questionnaire in order to minimize guessing and over or underestimating of PA levels, which may occur when patients fill in questionnaires on their own [55].

Additionally, the increase in frequency, duration and intensity of physical activity observed in this study was substantially larger than that reported in many previous PA-based behavioural interventions among CHD patients in either supervised or non-supervised PA programs [16, 27, 30, 54, 56]. An early study of supervised physical activity with CHD patients reported a greater increase in total moderate PA intensity and walking behaviour than we found here (supervised PA: 2058 METs/week; our study 886 METs/week) [54]. However, Heath and colleagues delivered a vigorous intensity physical activity intervention [57], and current guidance specifies that moderate PA (as we promoted) is clinically recommended for patients with CHD [58, 59]. The success of our intervention may in part be due to the implementation of a theoretically-driven intervention in which multiple behavioural change strategies were adopted. We encouraged patients to set personal tailored goals according to their needs; as such their goals were self-set, specific and personally relevant. Previous interventions had used generic ‘one-size-fits-all’ goals for their intervention participants [18, 27, 30–32, 34]. Our intervention included active self-monitoring strategies, in which participants were encouraged to record their activity levels in a diary, self-monitor their own progress and make their own plans to achieve their goals based on these observations. In previous studies, physical activity monitoring appeared to be more passive, involving the use of devices (such as accelerometers or pedometers) for automated recording of physical activity levels without participants taking responsibility for actively recording their behaviour or using the information to set tailored goals [27, 30, 31, 34].

Our intervention included regular tailored feedback for participants, which helped them to set their own goals; the combination of feedback with self-monitoring and goal-setting approaches had not been used in many previous PA interventions among CHD patients [22, 27, 30].

This intervention included regular follow-ups, individualised consultations and repeated reminders to act sent by text messages. Although the optimal frequency of follow-ups for PA behavioural interventions is unclear [60] it has been documented that frequent or intense contact between the participants and the health care providers increases the efficacy of PA interventions [16]. Although we do not have behaviour change outcomes beyond six months and as such cannot determine whether behavioural changes were sustained in the long-term, a prior intervention offering lower frequency of contact (2 contacts over 6 months) found a decrease in the use of self-regulation skills (goal-setting and self-monitoring) among patients six months after the intervention [27] suggesting that a lower frequency of contact may not result in sustained behavioural change.

The individualised consultations delivered within our intervention included feedback on progress, and tailored advice for patients on setting their own goals, and addressing their personal barriers to exercise. Studies with healthy participants have similarly shown that individualised, personalised consultations for the delivery of behavioural change strategies can successfully increase physical activity levels [61]. Text messaging was used in this study as a mechanism for reminding patients about their commitment to engage in regular PA, and to remind them of strategies that had been discussed to overcome their barriers. Prior research has suggested that using text messages for reminders to achieve goals enhances the mechanism through which implementation intention changes behaviour by improving the accessibility of plans [62].

The intervention improved health-related quality of life and this is consistent with previous PA interventions implemented together with other risk factor management interventions (diet, smoking, stress) in both supervised and home based PA programs among CHD patients [4, 16, 29, 63, 64]. Follow-up scores for health-related quality of life demonstrate that CHD patients in this study had improved health status and decreased impact of the disease following the intervention [65, 66]. This is an important finding since poor perceived quality of life may impact negatively on an individual’s ability to sustain behavioural changes in the long-term.

We observed significant reductions in blood pressure which has important implications for lifelong health. It is possible that patients may have altered their diet or smoking habits, or consumed antihypertensive medications prior to BP measurement. However, the reduction is likely to be associated with concurrent increases in physical activity, and this is consistent with previous studies among healthy people, hypertensive and CHD patients [67–69].

The intervention resulted in reductions in body mass index for CHD patients, and this has significant implications for the prevention or management of obesity (84.5 % of our sample were overweight or obese at the outset, compared with 72.7 % at follow-up), and for reducing the risk of co-morbidities. Improved body composition is likely to be associated with increases in physical activity, and improved BMI has been found in physical activity interventions elsewhere [52, 70, 71]. Prior studies have suggested that PA intervention may have little influence on body composition (e.g. body weight) when it is not used in conjunction with dietary intervention [2, 6, 72]. However, this study shows that intervention focused only on PA behaviour can generate clinically significant increases in reported PA activity together with reductions in BMI. This supports a review concluding that PA is associated with changes in body composition (e.g. weight loss) among overweight people even when it is implemented without dietary change methods [73].

Due to the nature of the physical activity intervention, it was not possible to blind participants or the nurse delivering the intervention to group allocation. However, it should be noted that the assessment of outcomes was not undertaken by an independent outcomes assessor, which may incur risk of bias and is a limitation of the study. However, the positive changes in objective variables (including body composition and blood pressure) may be perceived to be indicative of responsiveness to the intervention.

This study had a high participation rate, low attrition, high adherence and positive evaluation from intervention participants. Despite limitations of the study, it is possible that cultural factors may have played a role. Jordan is a developing country and as such, challenges within the healthcare systems that mean that individualised focus on lifestyle behaviours following cardiac incident is limited in scope. In these settings there is an absence of structured cardiac rehabilitation programmes, structured lifestyle advice and tailored intervention, whether as part of hospital and community care, or through research-delivered interventions. This absence of alternative options may have exerted an influence on patients’ willingness to participate and fully engage with the intervention, and as such the high rate of positive outcomes observed.

## Conclusion

This behavioural change intervention increased reported PA, improved health and psychological outcomes in CHD patients in Jordan. The intervention contained multiple elements, our magnitude of change was greater than that observed in previous studies, and all aspects of the intervention were perceived positively by participants. Through utilising multiple mechanisms for motivating behaviour change, this study addressed key limitations of prior research [10–12, 14, 15, 74] and we delivered an accessible, theory-based intervention in a setting where lifestyle interventions for CHD patients are limited, and inaccessibility is a common barrier to physical activity. Firm conclusions cannot be drawn from this study as to *which* elements of the intervention met with most success, and whether *all* elements of the intervention are required at the delivered intensity to generate the same magnitude of change. Further research is required to determine the ‘dosage’ of intervention required to generate physical activity behaviour change. Patients in this study were followed up only at six months and therefore it is not known whether these behavioural changes are sustained in the long-term. However, significant increases in self-efficacy were reported at six months (as found in previous PA interventions) [73, 75]. Increased self-efficacy is important since this intervention aimed to build self-efficacy for exercise, and as such, aimed to provide patients with lifelong skills to self-manage and sustain their own lifestyle behaviours over time. The cost-effectiveness of this intervention needs to be determined. There is scope for investigating pathways for implementing this intervention in new contexts. For example, different settings, as part of supervised PA programs, and/or in combination with other risk management programs (e.g. nutritional management).

### Summary illustrations

- Theory-driven, multicomponent behavioural intervention increases physical activity and improves body composition, health and psychological outcomes among patients with CHD who are not attending supervised PA programs.
- Research is needed to determine the optimal dosage and frequency of intervention required to generate clinically relevant behaviour change and health outcomes.
- Future studies should examine the contribution of individual behaviour change strategies in generating change, and the cost-effectiveness of the intervention.

## Abbreviations

BMI, body mass index; BP, blood pressure; CHD, coronary heart disease; CONSORT, consolidated statement of reporting trials; DPB, diastolic blood pressure; ESES, exercise self-efficacy scale; HRQL, health-related quality of life; IPAQ, International Physical Activity Questionnaire; ITT, intention-to-treat analysis; MCAR, missing completely at random; METs, metabolic equivalents; PA, physical activity; SBP, systolic blood pressure
